# Ideal personhood through the ages: tracing the genealogy of the modern concepts of wellbeing

**DOI:** 10.3389/fpsyg.2024.1494506

**Published:** 2024-11-12

**Authors:** Mohsen Joshanloo, Dan Weijers

**Affiliations:** ^1^Department of Psychology, Keimyung University, Daegu, Republic of Korea; ^2^Philosophy Programme, University of Waikato, Hamilton, Waikato, New Zealand

**Keywords:** wellbeing, authenticity, self-actualization, culture, ideal person, psychology

## Abstract

This inquiry traces the recent history of modern conceptualizations of personhood and wellbeing. It explores a general transition from traditional frameworks emphasizing social embeddedness, external obligations, and cosmic meaning to modern views privileging self-determination, authenticity, and self-expression. The inquiry shows that contemporary conceptions of wellbeing have emerged in Western cultures through the gradual accumulation of influences, including the Enlightenment ethos, liberal ideals, romanticism, existentialism, countercultural movements, and modern psychology. The important role of ‘authenticity’ is examined as a central value in contemporary wellbeing discourse, aligning with the modern conception of personhood. It is argued that contemporary perspectives tend to position the ideal of authentic self-fulfillment as an overarching paradigm that integrates self-determination, self-discovery, willful self-authoring, and creative identity expression. It is also argued that the widespread public support of the authentic self-actualization model in the contemporary era is partly due to the success of humanistic and positive psychology. This model’s prevalence is particularly notable in regions where the modern concepts of personhood prevail, i.e., in Western cultures and, to a somewhat lesser extent, within affluent non-Western cultures. By shedding light on the Western origins of modern concepts of wellbeing, this inquiry challenges their assumed universality.

## 1 Introduction

Some contemporary theories of wellbeing emphasize specific needs or values as universally significant across all individuals. However, a broader historical and cultural perspective reveals that modern scientific conceptualizations of wellbeing represent a distinct departure from most historical and traditional views. Assuming that these dominant notions of wellbeing are universally applicable risks oversimplifying the complex and culturally diverse ways that wellbeing has been understood throughout history and across cultures.

Wellbeing encapsulates an ideal state of existence for a Person. As such it is deeply intertwined with cultural and historical conceptions of personhood and the ‘ideal person’ (Kpanake, 2018; Sointu, 2012). This deep connection means that different historical and cultural perspectives of the ideal person have likely influenced how different conceptualizations of wellbeing have developed across the globe. To gain a richer understanding of contemporary conceptualizations of wellbeing, then, it could be valuable to explore the links between the development of conceptions of wellbeing and personhood through time and across cultures (Coan, 1977). Such an inquiry is undertaken here, describing aspects of the lineage underlying the dominant contemporary frameworks of wellbeing that play important roles in scientific, public, political, and social discourse today.

This inquiry examines the historical development of modern conceptualizations of ideal personhood and wellbeing, primarily in the context of Western cultures over the past several centuries.^1^ It distinguishes between traditional and modern perspectives^2^ on personhood and then examines the concepts of wellbeing associated with each perspective. After delineating these differences, the analysis turns its focus to ‘authenticity’ as a modern ideal that has become a cornerstone of contemporary wellbeing discourse. The changing status of authenticity, from a trivial concept to an ideal, is examined, shedding light on its ascendance in psychological theories of wellbeing. As the analysis unfolds, it will become evident that the emphasis on authenticity and self-actualization is an important aspect of many modern notions of personhood and may not align with most traditional perspectives on personhood. The significant role of psychology in integrating the ideal of authenticity into contemporary public culture is also highlighted, particularly through the important role authenticity plays in many modern conceptions of wellbeing. Critically, the inquiry emphasizes the necessity of acknowledging that modern conceptualizations of wellbeing tend not to resonate as strongly in more traditional, less globalized, and less affluent regions, where traditional notions of personhood are more prevalent. In doing so, this inquiry seeks to illuminate the Western, middle-class assumptions embedded in much of the contemporary wellbeing discourse and caution against presuming their cross-cultural supremacy.

## 2 The person in traditional worldviews

While neat chronological categorizations may not be entirely practical due to the constant evolution of the concepts of personhood over time, a broad distinction between ‘traditional’ and ‘modern’ perspectives serves as a useful framework for this inquiry. Generally speaking, traditional perspectives prevailed in societies before modernity and tended to conceive of personhood and wellbeing in ways that differ significantly from most modern conceptions (Cushman, 1996). In modern times the influence of traditional concepts has decreased, but they are still prevalent in some contemporary cultures and subcultures and present to a lesser degree in others.

In most traditional worldviews, individuals are not viewed as separate, isolated entities, but rather as integral parts of cohesive social groups (Cushman, 1996). On these views, one’s sense of identity emerges through active participation in collective social and cultural practices. This interconnectedness between individuals and their communities is viewed as a hallmark of both ancient and modern hunter-gatherer societies (Henrich, 2020; Moffett, 2019) and societies that relied primarily on hunting, gathering, and agriculture. In these societies, the individual self’s integration into the collective meant that individual wellbeing was intimately tied to the wellbeing and survival of the group (Belk, 1984; Morris, 1994). Best’s (1924, p. 398) account of New Zealand’s indigenous Māori people is a compelling example: “In studying Maori customs, it is well to bear in mind that a native so thoroughly identifies himself with his tribe that he always uses the first personal pronoun. In mentioning a battle that may have taken place ten generations ago, he will say, ‘I defeated the enemy there,’ mentioning the name of the tribe.” This illustration underscores how deep the sense of connection and unity between the individual and their community can go. This communal sense of personhood was dominant in all kinds of societies, from nomadic or sedentary hunter-gatherer societies to larger-scale settled societies, including village communities and urban civilizations that arose after the agricultural revolution; In these societies, personhood is still often seen as intertwined with a larger collective, such as kin, village, clan, or city. This traditional more collective sense of personhood persists in various non-Western regions of the contemporary world (Henrich, 2020; Joshanloo et al., 2021).

In much ancient scholarship, conceptions of ideal personhood emphasized finding one’s place in the cosmic order rather than self-expression and self-actualization.^3^ Many ancient philosophers and medieval thinkers viewed humans as fundamentally social beings, whose lives should be embedded in a larger social and moral framework (Melé and Cantón, 2014). This perspective emphasizes the importance of participating in a communal life that transcends individual interests and desires (Guignon, 2004). In addition, traditional religious ideologies often call for the suppression of individual selfishness in favor of participation in a divine or transcendent order. This notion of surrendering the self to a higher power was often seen as a means of achieving unity with the divine and other members of the religious community (Guignon, 2004; Martin and Barresi, 2006).

In most traditional frameworks, there are no firm boundaries between the inner realm of personal experience and the outer realm of public society and nature (Heelas, 1996; Rose, 1998; Taylor, 1992; Taylor, 1989). Without the more modern conception of separateness and psychological depth, there is little incentive to cultivate a distinct inner life of unique feelings and thoughts separate from communal roles and rituals. Rather than turning inward to discover an authentic inner self, the premodern self tended to be outwardly oriented and focused on fitting into external belief systems, traditions, and hierarchies that gave meaning to existence (Martin and McLellan, 2013). With strong distinctions between public appearance and inner truth mostly absent, personal identity tended to be expressed through participation in collective customs, rather than discovered in the inner core of the self. Internal or personal truths were often secondary to external obligations and sacred beliefs that situate the self in a broader sense of meaning and purpose. Indeed, individuals in many traditional cultures tend to discover and adopt the pre-existing meaning and purpose inherent in communal rituals, shared myths, prescribed duties, and societal roles (Cushman, 1990, 1996). On most traditional perspectives, the emphasis was on immersion in collectively shared systems of meaning, rather than the creation of unique personal meanings; meaning is less about shaping an individual life course and more about discovering one’s proper place in the sacred cosmic order posited by society (Baumeister, 2023). Traditional identity tended to depend on what one had to be publicly to be worthy of one’s assigned place in a cosmic or social order.

In sum, on most traditional perspectives, personal identity is intermingled with community, nature, and the cosmos rather than standing apart. Identity often came not from sincere expression of one’s unique and true self but from fulfilling responsibilities to the collective as a worthy, dignified member. Thus, the traditional person tended to be an intersection in a communal web rather than an autonomous, self-determining entity. For most traditional people, identity came from the acceptance of and immersion in a shared world rather than from autonomous choice and self-fashioning (Ho, 1995).

## 3 Traditional concepts of wellbeing

The conceptualization of wellbeing in any given era and context can be influenced by the cultural and social norms that define the ideal person in that context. Because of the differences between most traditional and modern conceptions of the individual, the understanding of wellbeing in most traditional societies differs in important ways from many modern conceptions. Not surprisingly, in many traditional societies, communal and spiritual concerns take precedence over the personal and subjective. In his investigation of the role of the sacred in human experience, Eliade (1987, p. 15; original emphasis) documents many examples in support of his claim that “[t]he man of the traditional societies is admittedly a *homo religious*.”^4^ Without the emphasis on an inner authentic self and psychological subjectivity that we find in many modern societies, wellbeing is more likely to be defined primarily by objective external standards instead of subjective feelings (Rose, 1998). Rather than prioritizing personal self-expression or the pursuit of individual desires, traditional cultures tended to focus on fulfilling assigned social roles to maintain communal cohesion and stability (Trueman, 2020).

On most traditional perspectives, therefore, wellbeing derived primarily from participating in collective rituals, following established traditions, and fulfilling familial and communal obligations, rather than focusing on developing and expressing a unique inner self and exercising independent agency. Maintaining orderly social dynamics and cosmic balance often took precedence over attending to and expressing personal and subjective feelings or desires (Baumeister, 1987; Cushman, 1996; Guignon, 2004). Adhering to the roles and responsibilities assigned within such traditional systems was crucial for avoiding social ostracism and maintaining public respect, prestige, and honor (Appell-Warren and Fong, 2014; MacDonald and Leary, 2005; Maner, 2017). On most traditional perspectives, deviating from communal norms was viewed as a sign of dysfunction, as it suggests disruption of the social or cosmic order that must be addressed through renewed engagement with the collective. Accordingly, individuals had to manage and regulate their subjective feelings and emotions to avoid disrupting their primary role as honorable contributing members of the larger community. This required individuals in these traditional societies to develop a sense of self-control and discipline and to learn to prioritize the needs and expectations of the community over their own personal desires and impulses (Markus and Kitayama, 1991).

On most traditional perspectives, the pursuit of personal happiness was often secondary to the fulfillment of social and cultural responsibilities, as the wellbeing of the individual was inseparably linked to the wellbeing of the community as a whole (Banda, 2019; Lacerda-Vandenborn, 2020). By actively participating in rituals, observing customs, and maintaining a harmonious social order, individuals in these traditional societies could achieve a sense of harmonious relation with a larger cosmic framework, which was considered the essence of wellbeing in most traditional societies. An example of this communal approach to wellbeing can be seen in Myers' (1979, p. 353) description of the Pintupi, an Indigenous Australian people who are one of the examples of a relatively modern-day society that has maintained this more traditional perspective:

The central themes of the Pintupi moral order revolve around the ideal of closely cooperating kin, and it is in terms of this understanding that Pintupi attempts to define when and how one should be ‘happy’ (*pukulpa*). Pintupi find it unusual that one could be ‘happy’ sitting alone; to be among kin, to be shown affection and concern, and to show it, should make one ‘happy.’ (Those who travel alone are suspect, and those who wish to be alone usually give some other reason.) While feeling ‘happy’ is an endopsychic matter-a ‘rising of the spirit,’ Pintupi seems to think that an individual experiences such states largely as the result of smoothly running relations between the individual and those he or she considers *walytja* [kin].

## 4 The person in modern worldviews

With the decline of feudalism, the rise of urbanization, and the modern market economy, a new conception of the person began to emerge in many societies (Cushman, 1996; Potter, 2011).^5^ This transformation did not happen overnight. It took place over centuries, beginning around the late Middle Ages and accelerating thereafter (note that determining its exact starting point is impossible). The way individuals tended to perceive themselves and their relationship to the collective changed with the establishment of modern cities and the growth of market economies (Guignon, 2004). The emergence of new social classes, increased mobility, and the decline of traditional social hierarchies all contributed to the development of a more individualistic and self-directed sense of identity in many people. The advent of new economic and social opportunities, coupled with the erosion of traditional social and meaning structures, often cultivated a more fluid and dynamic social environment. In this evolving landscape, individuals increasingly derived their sense of self from personal values, aspirations, and accomplishments rather than confining themselves to rigidly defined positions within static social hierarchies (Guignon, 2004).

Central to these new developments in many cases was the ideal of autonomy, namely, the capacity to make independent decisions and self-govern through reason, introspection, and personal choice (Rose, 1998). Contemporaneous with the ascension of liberal and humanist values during the Enlightenment, a new emphasis on determining and pursuing one’s own destiny and expressing one’s unique identity emerged (Martin and Barresi, 2006). Modern developments tended to highlight the significance of personal rights, liberty, and human dignity. At the core of many of these advancements was a novel concept of the person as a free agent who, through critical reflection and conscious choice, determines what constitutes a good life for themselves (Yin, 2018). This transformative period witnessed the emergence of a modern personal identity that directed individuals toward the pursuit of self-fulfillment rather than mere adherence to duties associated with religion, group affiliation, or the state (Martin and Barresi, 2006). For example, despite his belief in Heaven as the ideal human goal, Locke (1975) emphasized the importance of individual freedom to choose one’s path (although he hoped that everyone would choose to follow a religious one; Michalos and Weijers, 2017).

In many societies, this cultural transition marked a significant shift that prioritized the importance of internal subjective feelings over external obligations as a guiding force for moral choices and lifestyles (Rieff, 1987). This shift toward an internalized sense of self represents a significant departure from the common traditional concept of personhood, according to which individuals rely heavily on external sources such as community, tradition, and religious institutions for guidance, strength, and a sense of the sacred (Baumeister, 2023). In this emerging modern view, the sources of meaning and purpose turned inward, as individuals strive to nurture their own inner resources and gain a deeper understanding of their unique needs, qualities, and aspirations. This widespread transition from an external to an internal focus is often associated with the “massive subjective turn of modern culture, a new form of inwardness, in which we come to think of ourselves as beings with inner depths” (Taylor, 1992, p. 26). Rieff (1987) notes that this subjective turn coincides with the emergence of the ‘psychological person,’ characterized by a heightened sense of individuality, a greater acknowledgment of the inner life, and a tendency to prioritize personal experiences and fulfillment over external influences. Indeed, many people now find meaning in their subjective experiences (Hicks and King, 2009). And, many others perceive themselves as creators of personal meaning through intellectual autonomy, creativity, and personal choices (Santiago, 2005).

It is noteworthy that we do not argue that subjectivity and introspection are peculiar to modernity. Premodern societies undoubtedly possessed a sense of inner experience. However, the intensification of subjectivity in recent centuries, catalyzed by advances in survival, security, and individual freedoms, has made it a defining feature of modern existence in many societies. As a result, many contemporary individuals have internalized the locus of meaning, spirituality, and self-evaluation, seeking answers to existential questions within rather than without. This paradigmatic shift underscores the centrality of subjectivity in modern life and distinguishes it from premodern configurations of self and society that were highly attuned to broader communal and spiritual contexts.

## 5 Modern concepts of wellbeing

The psychological literature provides a useful source for exploring contemporary conceptions of personhood and wellbeing (Teo, 2018). Psychological models of wellbeing are influenced by both culturally specific theoretical frameworks advocated by psychologists and empirical data collected from laypeople. As social scientists, psychologists strive to reconcile theories with the empirical realities of individual experience. This involves refining or even rejecting theoretical perspectives that mismatch empirical data from various sources (Howitt and Cramer, 2020). While acknowledging the limitations of empirical model testing (Preacher and Merkle, 2012), psychology’s blending of theory and data lends some credibility to its insights into modern wellbeing concepts. Furthermore, the subjective turn in modern society, emphasizing inner experience (Rieff, 1987; Sarauw et al., 2023; Taylor, 1992), coupled with psychology’s expertise in understanding inner experiences, positions the field to offer useful insights into contemporary perspectives on wellbeing. Finally, psychology’s focus on both optimal and non-optimal human functioning (Joshanloo, 2021) makes it a highly relevant source of knowledge for understanding modern conceptions of wellbeing. In sum, although psychology does not have exclusive authority over modern conceptions of wellbeing, an examination of psychological models of wellbeing can shed light on key aspects of the contemporary conceptualization of wellbeing.

To better understand the key elements of wellbeing emphasized in contemporary psychology, we can examine the central themes that emerge from a review of prevalent psychological models of wellbeing (e.g., Diener et al., 1998, 2010; Lyubomirsky and Lepper, 1999; Martela, 2023; Ryan and Ryan, 2019; Ryan and Vansteenkiste, 2023; Ryff, 1989; Ryff and Singer, 2008; Waterman et al., 2006, 2010). The following is a non-exhaustive list of central themes emphasized in these models:

- Autonomy: Being able to make decisions and think independently, guided by one’s own values and preferences, and free from external pressures or limitations.
- Competence/environmental mastery: The ability to shape one’s surroundings, influence one’s circumstances, and achieve outcomes one desires.
- Self-expressiveness: Expressing one’s true self through one’s beliefs, behaviors, and choices.
- Meaning and purpose: Discovering meaning in one’s life experiences and having personal goals that give one a personal sense of purpose and direction in life.
- Self-acceptance and self-esteem: Embracing all aspects of oneself, both the positive and negative, and having a positive self-evaluation and sense of worth based on one’s own internal standards.
- Good relationships: Building and nurturing fulfilling connections with others while also maintaining one’s individuality and personal boundaries.

Clearly, these interconnected and overlapping themes align well with common modern understandings of ideal personhood and differ from most traditional perspectives. It is well known that the majority of influential psychological research is based in the West and especially in the United States (Cheon et al., 2020). This is also true for wellbeing-focused psychological research, including positive psychological interventions (Hendriks et al., 2019). Many of the influential modern models of wellbeing, such as those mentioned above, are no different—they are based on mainly Western concepts, conducted by mainly Western researchers, and validated mainly using participants from the West. This suggests that modern conceptualizations of wellbeing may be culturally biased (Christopher, 1999; Joshanloo et al., 2021). Most traditional views of optimal personhood and wellbeing emphasize the importance of belonging, conforming to group norms, adapting to external circumstances, living in harmony with others and nature, and prioritizing communal needs over individual desires (Joshanloo et al., 2021). On such traditional views, individuals often find their sense of wellbeing through harmonious relationships with family, community, and established values. These views tend to focus on the fulfilment of obligations to the community and finding a balance between personal aspirations and the needs of the group. As such, personal wellbeing is closely tied to collective wellbeing, and a fulfilling life is often associated with achieving a respected social status within one’s community. This status is usually achieved by dutifully fulfilling obligations, maintaining harmonious relationships, and observing established norms and customs. Most traditional concepts of wellbeing prioritized connectedness over individuality, social responsibility over personal autonomy, conformity over self-expression, and communal wellbeing over personal happiness. Importantly for the following discussion, most modern concepts of wellbeing do the opposite, emphasizing key elements of authenticity: individuality, personal autonomy, self-expression, and the satisfaction of individual desires.

### 5.1 Beyond binaries: co-existence of traditional and contemporary personhood

Personhood is not uniformly defined within any specific culture, and the transition to a more modern conception emphasizing individual freedom and autonomy has proceeded at varying paces worldwide. Compared to their non-Western counterparts, Western cultures have experienced more significant shaping influences from modernity, including the Enlightenment, the emergence of liberal democracies, and the accumulation of wealth (Kirkpatrick, 2014). Consequently, Western societies are often seen as forerunners in developing modern ideas about personhood and wellbeing. However, traditional community-oriented social structures persist in the West, particularly among certain groups (e.g., some immigrants, religious minorities, and isolated villages). For example, Iyengar and Lepper (1999) found that choosing for themselves promoted intrinsic motivation, enjoyment, and success for Anglo American children, but not for Asian American children, who tended to accrue these wellbeing-related benefits when decisions were made for them by their mothers or their in-group. This underscores the ongoing coexistence of both traditional and modern conceptions of personhood and wellbeing within Western cultures. Nonetheless, at this juncture, modern conceptualizations appear to predominate in the West (Christopher, 1999; Joshanloo et al., 2021).

The non-Western world is more diverse. Affluent East Asian societies, for example, have experienced rapid economic growth and technological advances that have led to increased individual expression and autonomy, mirroring certain aspects of Western modernity (Kyung-Sup, 2014). However, Confucian ideals of social harmony and filial piety remain deeply influential, fostering a unique brand of modernity (Liu, 2021). Conversely, certain cultures, particularly in Africa and the Middle East, may seem slower to adopt the dominant modern Western concepts of the person. This reflects not only resistance but also the continuing influence of established traditional structures. Clan affiliation, religious doctrine, and reverence for ancestors continue to play a central role in shaping personal identity in many societies in these regions, influencing societal norms and individual aspirations. In general, traditional notions of personhood and wellbeing are more prevalent in non-Western countries than in Western countries (Joshanloo et al., 2021). However, as non-Western societies advance in their levels of development and wealth, modern notions of personhood and wellbeing are becoming more prevalent in non-Western cultural contexts as well. Therefore, all cultures, without exception, experience the coexistence of both traditional and modern elements to some degree.

## 6 Authenticity

Authenticity refers to being true to one’s genuine self by knowing and consistently expressing one’s genuine values, beliefs, and emotions in one’s daily life (Guignon, 2008). We argue that the ideal of authenticity has become a pervasive and deeply ingrained notion in contemporary society, encompassing a multifaceted concept that combines elements of essentialist, existentialist, and expressive individualist thought. At its core, the ideal of authenticity posits the existence of a true inner self that lies beneath the surface of external influences and social conditioning. This authentic self is believed to be a unique and valuable entity that must be discovered, nurtured, and expressed in order to achieve a sense of true identity and fulfillment. This true self is not considered fixed or predetermined, but rather can be self-created, self-authored, and self-transformed through individual choice and agency. In other words, authenticity is not just about discovering one’s essence, but also about creating and recreating oneself through an ongoing process of self-reflection, self-expression, and self-reinvention. Furthermore, the ideal of authenticity emphasizes the importance of protecting this true self from the corrosive effects of external forces, such as societal expectations and traditional norms, which may be seen as threatening to obscure or distort one’s authentic nature. By embracing this ideal, individuals are encouraged to embark on a journey of self-discovery, to shed the masks of conformity and to freely express their true selves, thereby realizing their full potential and living a more authentic, meaningful life.

In the subsequent sections, we will further deconstruct the various components of our argument and provide a concise historical account of the concept of authenticity, tracing its development from its early modern roots to its contemporary manifestations. This historical analysis will reveal how the normative status of authenticity has shifted dramatically in many societies, from being considered a trivial pursuit to becoming an idealized and central aspect of theories of wellbeing, laying the groundwork for its current prominence in modern cultural discourse. By examining the evolution of authenticity through recent history, we will illuminate the ways in which many contemporary formulations of wellbeing have taken shape. We will then contend that, despite its widespread cultural significance, the modern notion of authenticity may not resonate with individuals who adhere to more traditional values and ways of life.

### 6.1 A break from traditional times

Trilling (2009) traces the origins of the contemporary emphasis on authenticity to the historical primacy of sincerity as a virtue. The value of sincerity emerged around the late 16th and early 17th centuries as a reaction to the perceived artificiality and hypocrisy that had become prevalent in contemporary societies. The emphasis on sincerity represented a break from the conventional social virtues in most societies, which emphasized strict conformity, propriety, emotional restraint, and conscientiousness. The virtue of sincerity emphasizes the correspondence between one’s outward appearance and one’s inner nature. This ideal places great emphasis on personal honesty and integrity in one’s words and actions. Trilling (2009) argues that, over time and as social values changed, a more complex understanding of authenticity emerged that went beyond mere sincerity. Sincerity can be understood as being true to others. The evolving concept of authenticity, in contrast, emphasizes the articulation and affirmation of one’s deepest personal identity; that is, an innate core self waiting to be discovered and expressed within each individual (Guignon, 2004; Trilling, 2009).

The confluence of developments in much modern scientific, religious, and philosophical thought catalyzed the historical shift from conventional social virtues to the emerging ideal of authenticity in many societies (Lindholm, 2013; Potter, 2011). Developments in Protestant religious thought brought a greater emphasis on introspection, self-evaluation, and personal relationships with the divine, practices conducive to the later development of sincerity and authenticity as ideals (Lindholm, 2013). Similarly, modern philosophical thought increasingly discussed notions of human freedom, autonomy, and the capacity for self-determination and self-reliance (Varga, 2013). Against this modern backdrop, authenticity gained traction by offering an alternative paradigm of virtues that shifted inward and emphasized uncovering one’s subjective depths and expressing them openly. If common traditional virtues prioritize social cohesion secured by duty and propriety, the new virtues privilege understanding and articulating one’s innate individual identity as a source for determining moral thought and action. The ideal of authenticity, however, did not attain widespread prominence until the romantic and existentialist movements of the 18th to 20th centuries chose authentic self-expression as a central value (Lindholm, 2013; Varga, 2013).

### 6.2 Two types of authenticity: essentialist and existentialist

What is typically called the ‘essentialist’ or the ‘inner sense’ model of authenticity emerged in 18th-century European thought, pioneered by Jean-Jacques Rousseau (Lindholm, 2013; Varga, 2013). Though Rousseau did not explicitly use the term authenticity, his works introduced the notion of finding and living by one’s innate true self as crucial for a good life. Rousseau (1754, 1964) depicted this true self as rooted in humanity’s natural goodness, but corrupted by society. As such, he prescribed a return to nature and introspective self-examination to reconnect with one’s authentic core. Rousseau’s ideas inspired subsequent Romantics to develop this concept of authenticity further. The specific terminology of authenticity became attached to the romantic school of thought in later centuries (Leuenberger, 2021). The essentialist model of authenticity posits that every individual possesses a unique and immutable inner essence, often referred to as the ‘true self’. Authenticity, in this perspective, usually entails a journey of self-exploration to uncover this inherent essence, which is often hidden by cultural expectations, and align one’s actions and identity with it. Failure to achieve this alignment is seen as resulting in inauthenticity, a sense of alienation, and a loss of personal identity.

Another model of authenticity, the ‘existentialist’ or ‘productionist’ model that emerged later, denies a pre-existing true self. It sees the authentic self as freely created through one’s choices and actions (Iftode et al., 2023; Varga, 2013). Sartre and de Beauvoir, expanded on ideas from Kierkegaard, Heidegger, and others to fully articulate an existentialist concept of authenticity in the mid-20th century (Panza and Gale, 2008). The existentialist ideal of authenticity emphasizes radical freedom and self-creation as opposed to submitting to conventional identities and roles. It encourages questioning established beliefs and values to empower individuals to develop an authentic perspective driven by their inner motivations. It urges actively defining one’s own relationship to existence through responsible self-authorship rather than passively accepting dominant cultural interpretations. Overall, the existentialist conception of authenticity focuses on individual sovereignty in shaping one’s identity and life (McCann, 2011; Panza and Gale, 2008).

While there are similarities, the distinction between the two types of authenticity can be summarized as follows. Essentialist authenticity sees the true self as innate, pre-existing, and waiting to be discovered and expressed. It defines authenticity as the faithful expression of this pre-given nature. Any changes or deviations reflect contaminating external influences that mask or corrupt one’s innate core. In contrast, existentialist authenticity rejects any pre-defined essence and sees the individual as radically free to shape his or her own nature. Existentialists deny any pre-defined inner core that constitutes the true self. For them, authenticity consists of directly exercising this freedom to shape oneself through one’s free choices. Letting external factors dictate one’s direction represents inauthenticity. In sum, romanticism links authenticity to the discovery and manifestation of a true self, while existentialism links it to self-creation. A crucial point is that essentialist and existentialist authenticity, while seemingly divergent, have substantial overlaps that intertwine them in contemporary thought (Iftode et al., 2023). Essentialism’s notion of finding one’s inner, essential self cannot be divorced from an act of self-creation in choosing to search for and interpret this supposedly true identity in order to express and live it out. Similarly, existentialism’s emphasis on radical self-authorship cannot occur in a vacuum; it must be based on some seeds of innate proclivities that shape the will to invent oneself in the chosen way rather than other ways.

The idea of many traditional views of the self, to which the romantic and existentialist schools responded, is that one’s identity and life path are largely (and appropriately) determined by external factors such as social background, gender roles, family occupation, religion, and so on (Baumeister, 2023). In these traditional worldviews, the self was not seen as something to be independently discovered, defined, or expressed. Rather, personal identity was conferred by one’s position within an ordered cosmos and social order that was seen as natural, divinely ordained, and unquestionable (Guignon, 2004). For many in these times, life trajectories were thus fairly rigid and prescribed at birth according to status and gender (Baumeister, 1987, 2023). Personal preferences and inner potentials were largely irrelevant compared to birthright duties and constraints in directing one’s life course. Freedom in self-definition outside of these strong communal and religious bonds and expectations was often not considered ideal or even possible. Traditional Christian (Drever, 2017) and Muslim/Sufi (Ahmadi and Ahmadi, 1998) writings often encouraged self-reflection, but with the ultimate goal of fostering a connection with the divine within rather than merely recognizing and expressing one’s unique self.^6^ In other words, the promoted self-examination did not usually aim at the discovery of an innate inner essence that defined one’s individuality, but rather sought to transcend individuality by uniting with the universal divine essence, which can involve a renunciation of one’s sense of individuality (Ahmadi and Ahmadi, 1998).

On most traditional perspectives, social order and culture were imbued with a sense of sacredness, and it was assumed that one’s wellbeing was inextricably linked to finding one’s rightful place within the established conventional framework. This approach required individuals to adjust or suppress any inner desires to resist conforming to cultural and social conventions and norms. However, the emergence of the new paradigm of authenticity challenged this traditional view. Within this paradigm shift, the external world and cultural conventions were perceived as potentially corrupting influences, and the locus of wellbeing shifted inward. The preservation of one’s inner self against the perceived corrupting influence of society and the cultivation of one’s true self emerged as paramount to wellbeing in many societies. This shift in perspective underscored the importance of self-discovery and self-actualization, redirecting the path to wellbeing for many people from external conformity to internal exploration and self-realization (Cushman, 1996; Taylor, 1992; Varga, 2013). This inward focus led to a new reverence for the depths of human interiority—a sense of amazement at the mysterious complexity and sacred potential of our inner lives and subjective experience. Increasingly, the sacred was no longer found outside but inside.

However, adhering to the ideal of authenticity encompassed more than merely uncovering or creating one’s true self. An important aspect of the ideal of authenticity is its emphasis on *expressing* one’s true or self-authored self in one’s outward actions and lifestyle. This expressive dimension of authenticity is linked to ‘expressive individualism’ (Bellah, 1985; Cortois and Laermans, 2017; Trueman, 2020), which is another modern value emphasized in much of the contemporary Western world. Expressive individualism emphasizes the exploration, expression, and fulfillment of one’s inner potential and desires. It advocates for a society that enables safe self-discovery and self-expression across various aspects of life. According to expressive individualism, living an admirable and authentic life involves expressing and outwardly demonstrating our deepest personal visions and talents, rather than conforming to external norms and expectations.

To summarize, in today’s world, the dominant concept of authenticity combines elements of essentialist authenticity, existentialist authenticity, and expressive individualism. Grounded in the fundamental principles of modernity (e.g., autonomy, freedom, and humanism) and the modern concept of personhood, the quest for authenticity entails uncovering our true selves through introspective self-examination, actively molding this self through intentional choices, and creatively expressing it in our outward lives. As traditional religious beliefs declined in modern society, individuals turned inward in their search for purpose, meaning, and self-understanding. As long-standing social conventions and religious tenets that previously imbued life with purpose diminished in modern times, many individuals increasingly came to see these traditional paradigms as stifling doctrines that narrowly prescribed ways of being, thinking and behaving, thereby hindering the capacity to live freely and realize an authentic self.

### 6.3 Authenticity and psychology

Psychology emerged as a formal academic discipline in the late 19th century. During the mid-20th century, two major theoretical frameworks dominated the field: psychodynamic theory and behaviorism (Schultz, 2013). Psychodynamics contributed to the formulation and development of concepts related to human interiority and inner depth within psychological and public discourses. This perspective popularized the concept of a ‘real self’ that transcends the superficialities of conventional social interactions. It emphasized the importance of self-exploration and the expression of unconscious drives as a means of uncovering and embracing one’s true self (Ellenberger, 1970; Illouz, 2008; Guignon, 2004). Hence, psychodynamic concepts aligned with the essentialist model of authenticity. Psychodynamics also underscored the pivotal role of the conscious rational ego in shaping or reshaping personality and behavior, as well as mediating between the inner self and external reality (Illouz, 2008), thereby resonating with the existentialist model of authenticity. However, authenticity was not a primary focus within classical psychodynamic theory (Thompson, 2005). Behaviorism, in contrast, deliberately avoided the exploration of internal subjective states, concentrating exclusively on external stimuli and observable behavior. By locating the source of behavior in the external environment, behaviorism effectively diminished the role of the inner self in shaping one’s actions and experiences, suggesting instead that the self is largely a product of external influences. Despite their differences, both schools upheld a fundamental determinism regarding human behavior, attributing causality to irrational drives, childhood experiences, or environmental factors (Scalambrino, 2018).

Humanistic psychology emerged in the mid-20th century partly in response to the deterministic and mechanistic theories that dominated psychology at the time, namely psychodynamics and behaviorism. Led by influential figures like Abraham Maslow, humanistic psychologists recognized the need for a more holistic, human-centered ‘third force’ in psychology (deCarvalho, 1990). Given its strong ties to existential philosophy (Winston, 2015), it is not surprising that the concept of authenticity, which plays a pivotal role in existential thought, became a central tenet in humanistic psychology as well (deCarvalho, 1990; Medlock, 2012).

A key contribution of humanistic psychology was the deployment of the organismic model of human behavior and development to challenge the mechanistic model favored by psychodynamics and behaviorism (Morley, 1995). The organismic model views humans as inherently active agents who give meaning and organization to their own behaviors and experiences, rather than being passively controlled by external forces (Lerner, 2018). This perspective sees change and development as intrinsic to human nature because of our drive to integrate experiences into a meaningful whole. Therefore, from an organismic perspective, development in any domain emerges from within the individual as a purposeful process of self-organization, rather than resulting solely from efficient or material causes in the external world. While environmental factors may facilitate or constrain growth, the formal basis for development according to the organismic model is the overall structure and configuration of the individual’s mental and behavioral life. The organismic perspective includes a teleological element, meaning that development is directed toward an ultimate goal or end state (Goldstein, 1934). This developmental ideal pulls the individual toward the fully realized form they are destined to take (Lerner, 2018; Reese and Overton, 1970).

Working within the organismic framework and based on a modern conceptualization of personhood, humanistic psychologists defined mental wellbeing in terms of self-actualization—the innate human need to grow and become all that one is capable of becoming (deCarvalho, 1990). Self-actualization represents the highest point of human development, upon which we fully realize our unique potential. It involves gaining a deep understanding of our authentic selves, our values, talents, and purpose, and having the courage to authentically express who we are to the world. Authenticity allows us to create a personalized path that nurtures our individual gifts and passions. Therefore, self-actualization requires living in alignment with our true selves, as deference to external authorities can hinder our personal growth. In essence, honoring our true identity empowers us to express our unique inner greatness, which is critical to achieving self-actualization (Illouz, 2008). In this way, the idea of self-actualization connects back to the romantic and existential notion of authenticity. Living authentically means staying true to who you are and manifesting your innate dispositional potential rather than conforming to societal roles or expectations.

Although humanistic psychology soon lost prominence as an established field, its theoretical impact on psychological discourse was substantial and enduring (Elkins, 2008). Humanistic psychology sparked a significant ‘humanistic revolution’ within psychology (DeRobertis, 2021). The organismic perspective it championed has largely displaced mechanistic models across modern psychology. For instance, self-determination theory, one of today’s most influential motivational frameworks, is grounded in an organismic view of human agency and growth tendencies and emphasizes values such as autonomy, self-determination, and authenticity (Ryan and Deci, 2019; Ryan and Ryan, 2019). Terms like self-actualization have become thoroughly ingrained in the lexicon of mental health (Illouz, 2008). Furthermore, the humanistic formulation of authentic living and self-actualization provided the foundation for contemporary scientific and popular conceptualizations of wellbeing. Humanistic psychology’s influential conceptualization of wellbeing as authentic self-actualization continues to shape modern perspectives on wellbeing.

Positive psychology, currently the most visible psychological approach to wellbeing, inherited humanism’s organismic assumptions and emphasis on authentic self-expression and self-fulfillment as the essence of wellbeing. Although contemporary psychologists may use alternative terminology (e.g., eudaimonic wellbeing) to describe wellbeing, the underlying concept shares significant parallels with the humanistic perspective (Robbins, 2008). As Medlock (2012, p. 38) points out, for both humanistic and positive psychology “an ethic of authenticity provides a unifying normative framework.” Medlock explains that the ethic of authenticity is a normative framework that emphasizes the importance of living in accordance with one’s true self. It involves a process of self-discovery, in which individuals clarify their values, preferences, and interests, and make choices and commitments that align with their authentic selves. Thus, while humanistic psychology failed to sustain momentum as an organized movement, its conceptual assumptions are now a central part of mainstream psychological wisdom (Elkins, 2008).

An examination of the core themes in psychological models of wellbeing, as previously reviewed, reveals a strong resonance with the ideal of authenticity. Key concepts such as autonomy, competence, self-expression, meaning, self-acceptance, and meaningful relationships all play a fundamental role in enabling us to live authentically towards the ultimate goal of self-actualization. For example, autonomy gives us the freedom to determine our own path according to our personal values, enabling us to make authentic choices. Competence empowers us to navigate our environments effectively, shaping a life and lifestyle based on our own standards. Self-expressiveness allows us to outwardly manifest our inner truth. Discovering personal meaning and purpose aligns our actions and life direction with our authentic beliefs. Self-acceptance and self-esteem allow us to embrace our complete selves, including our imperfections, as the basis for personal development. Lastly, meaningful relationships require us to reveal authentic aspects of ourselves and honor the authentic expressions of others. In essence, these central themes that modern psychology emphasizes all contribute to the exploration, cultivation, and manifestation of our authentic selves with the ultimate goal of self-actualization. Thus, contemporary theories of wellbeing tend to emphasize authenticity as an overarching ideal.

### 6.4 Contemporary public discourse

Modern cultural currents such as liberal humanism, romanticism, and existentialism have joined forces to create the ideals of authentic self-expression, autonomy, and self-actualization in contemporary public discourses on wellbeing (Madsen, 2015). Twentieth-century countercultural movements also brought a heightened focus on challenging societal constraints and manifesting innate human potential (Burnett, 2011; Heelas, 1996). Humanistic psychology, inspired and influenced by all these cultural undercurrents, produced an intuitive and persuasive conceptualization of wellbeing centered on realizing one’s true self, facilitated by individuality and nonconformity. This conceptualization powerfully resonated with many people today, promising a conception of self-realization that sidestepped restrictive social conventions. The widespread adoption of humanistic principles, emphasizing inherent human agency and the drive for personal growth, contributed to the integration of these concepts into public discourse. Similarly, the positive psychology movement carried on the humanistic legacy, adding more empirical and scientific credibility to its focus on authentic living, self-determination, and self-actualization. The public embrace of positive psychology as a scientific guide to self-improvement reveals a lingering cultural attraction to humanistic psychology’s original promise of realizing wellbeing through authentic self-fulfillment.

The influence of this legacy persists in current public discourses, particularly among contemporary middle-class populations in Western societies, but also in more affluent and democratic regions of the non-Western world (albeit to a weaker degree). In such environments, living an authentic life is often regarded as a fundamental component of leading a fulfilling and meaningful existence (Madsen, 2015; Potter, 2011; Sointu, 2012). The authentic person demonstrates fidelity to an inner essence that defines individuality, while exercising freedom to shape life direction unencumbered by conformist pressures (Illouz, 2008). Fulfillment is realized through embarking on a personalized journey of self-actualization that channels innate potential toward passionate self-expression. This weaving together of self-discovery, self-creation, and self-expression captures the modern striving for authenticity. The pursuit of authenticity entails an intense commitment to self-determination that values each individual’s distinctive approach to life. The middle-class citizens of modern nation-states are characterized by their relentless pursuit of personal fulfillment by embracing their unique paths (Burnett, 2011; Cabanas and Illouz, 2019).

Many contemporary individuals often take the ideal of authenticity for granted, perceiving it as a natural and intuitive roadmap for living well (Potter, 2011). This perspective is particularly prevalent in urban areas, where popular culture, media, and psychological discourses frequently advocate for leading an authentic life. Common expressions like ‘Stay true to who you are!’, ‘Embrace your true nature!’, ‘Do not pretend to be someone you are not!’, ‘Be yourself!’, ‘Listen to your inner voice!’, ‘Live your truth!’, and ‘Find your own path!’ underscore the societal emphasis on authenticity. However, it is important to recognize that this contemporary understanding of authenticity as a key to wellbeing has evolved over recent centuries and contrasts sharply with traditional values of conformity, propriety, restraint, devotion, dutifulness, and even self-abnegation. These contemporary imperatives that encourage individuals to prioritize self-expression and personal fulfillment over societal expectations, may not be relevant for individuals who adhere to traditional standards of living. While these values are not always irrelevant or incorrect in the context of traditional cultures, such cultures often prioritize more collectivistic considerations over self-centered concerns when conceptualizing wellbeing. From their perspective, these concerns might be perceived as self-centered, immature, or potentially disruptive to the social order, at least on some occasions.

## 7 Conclusion

This inquiry unveils some of the historical roots shaping modern conceptualizations of wellbeing. By exploring these roots, this inquiry demonstrates how modern conceptualizations of wellbeing, centered on the ideal of authentic self-actualization, have evolved over the past few centuries, influenced by a diverse array of cultural elements predominantly drawn from the Western world. The emergence of this new paradigm of authenticity is linked to a new conception of the person that developed within the modern era, with increased urbanization, mobility, and affluence. The romantic and existentialist movements further solidified authenticity as a central virtue guiding the individual’s quest for a meaningful and fulfilling life. This modern perspective emphasizes individual freedom, the primacy of subjective experience, self-creation, and resistance to social influence.

The modern framework significantly departs from most traditional worldviews, which ground identity and purpose in communal bonds, cosmic orders, and external obligations. Many traditional frameworks seek to mitigate self-centered concerns by emphasizing communal responsibilities and/or religious principles to foster social harmony and cohesion. These frameworks legitimize conformity to external authority and the suppression of personal desires. In contrast, modernity brought about a radical shift that prioritized the inner self as a sacred source of truth and guidance. The advent of modernity led to the increasing recognition, exploration, and expression of personal emotions, values, and insights as a guiding source of lifestyle choices and validation. The inner realm is routinely sanctified as the repository of authentic insights for charting one’s unique path, with social and religious constraints increasingly perceived as oppressive barriers to true freedom and authentic self-expression. Many contemporary discourses align with modern conceptualizations of personhood and wellbeing, promoting a lifestyle that underscores the significance of introspection, self-authoring, and living in harmony with one’s inner truths. Much psychological discourse has played a significant role in promoting and legitimizing this new framework by providing empirical evidence to support its premises and lending it the authority of scientific research. As a result, dominant parts of psychology have contributed to reinforcing the prevailing cultural belief in the importance of authenticity and self-expression in public discourse.

This inquiry into the historical trajectory of the concept of personhood and the ideal of authenticity reveals that the prevailing conceptualizations of wellbeing, rooted in the pursuit of authentic inner potentials, are not necessarily natural or universally applicable. The promotion of authentic self-actualization as the foundation of wellbeing reflects the modernist focus on the inner self rather than external authorities. These formulations have emerged from a convergence of influences drawn from mainly Western traditions, such as Protestantism, the Enlightenment, humanism, capitalist economic structures, progressive countercultural movements, and modern psychology. This inquiry highlights the evolution of interconnected notions of personal autonomy, subjective interiority, and authenticity over centuries, shaped by unique paradigm shifts in predominantly European and North American contexts. Therefore, it becomes evident that ideals and values such as self-determination, self-definition, and self-fulfillment are not universal or innate concepts. Instead, they embody cultural assumptions that are in line with Western middle-class ideals, such as independence, mobility, liberation from assigned roles, a diversity of choices and lifestyles, and the celebration of individual freedom, creativity, and expression. These ideals often conflict with more traditional structures that prioritize social interconnectedness, obligations to family and institutions, customary rituals, and conformity to collective norms and beliefs. The concept of wellbeing, based on the realization of subjectively meaningful potentials, assumes levels of personal freedom and objective structures and realities that may be inaccessible to contemporary individuals in some cultures aligned with more traditional values. This is particularly true when people in some of these more traditional cultures are constrained by restrictive circumstances or preoccupied with securing basic survival needs.

Therefore, presenting authentic self-actualization as the universal ultimate goal of human wellbeing risks obscuring its origins within Western culture. The importance of imperatives such as ‘Be yourself!’ should not be separated from the cultural context that influenced these values. This inquiry aims to contextualize the prevailing authenticity-based paradigms by tracing their formative influences. It serves as a reminder to exercise caution when treating these constructs as universal truths. By shedding light on the relatively recent development of these standards, this research urges caution in assuming the inherent dominance of modern frameworks over traditional ones. Traditional frameworks continue to exert influence on a global scale, even among some people and subgroups within Western cultures, highlighting the need to acknowledge the diverse cultural contexts that exist worldwide, especially in descriptive research. By shedding light on the Western origins of modern ideals, this inquiry warns against imposing authenticity-based wellbeing concepts as a universal standard, particularly when applied to individuals or cultures that uphold more traditional values and customs. Imposing such constructs risks perpetuating cultural insensitivity and ignorance and failing to accurately measure wellbeing from at least some people’s perspective.

## Data Availability

The original contributions presented in the study are included in the article/supplementary material, further inquiries can be directed to the corresponding author.

## Footnotes

^1^This inquiry focuses on Western history and cultures because they have likely had the most influence on current Western conceptions of wellbeing. There have certainly been some cross-cultural influences over the centuries, and some important instances are mentioned below, but a detailed account of these is avoided in order to focus on the main argument.

^2^We use the terms “traditional” and “modern” to indicate that modern views emerged after traditional ones. We do not consider either “traditional” or “modern” views as inherently superior; the distinction is purely chronological. We acknowledge the considerable diversity within both traditional and modern views. We also acknowledge that modern views can be traced back to ancient times and that traditional views can be found in modern times. We use these general terms to distinguish two broad categories of views that, from our perspective, are qualitatively different from one another at a conceptual level, despite the variability within each category, and that were more common in different eras.

^3^Of course, there were also ancient philosophers that argued for a more individualistic perspective or a balance between the two. Aristotle, for example, argued for the importance of both political engagement and introspective philosophizing to develop the virtues and live an ideal life (Danford, 2000). It should be noted that the more individualistic philosophers were arguing against the cultural mores of their time. Additionally, ancient accounts often share a common underlying theme of communal existence and a deep connection to the cosmos, as well as an understanding of life as part of a spiritual order. Calls for self-reflection in these traditions should thus be interpreted within the broader context of this spiritual and communal framework, where individual introspection is intertwined with one’s role in a larger, interconnected reality. Here, we argue that in modern calls for authenticity, authenticity itself is viewed as an end rather than a means. Self-reflection, self-discovery, and self-expression are pursued not for purposes dictated by a conventional communal or spiritual system of beliefs, but rather for their intrinsic value to the individual within a modern, often secular, context.

^4^We thank an anonymous reviewer for suggesting the work of Mircea Eliade.

^5^As mentioned earlier, the roots of a more individualistic view of the person can be traced back further than this, but this is when general opinion, culture, and lifestyle (rather than small intellectual developments) began to change across many societies.

^6^The exhortations of modern Sufi, Buddhist, Hindu, and other holistic spiritual writers to embark on introspective journeys and cultivate self-awareness must be situated within the broader context of a spirituality that embodies the fundamental interdependence of all existence (Joshanloo, 2014). In these traditions, the human experience is often metaphorically likened to a droplet of water in the vast ocean of spiritual reality, underscoring the imperative of transcending the ego’s boundaries and dissolving the dichotomy between the individual self and the larger cosmos. The ultimate aspiration is not to crystallize the self as a discrete, autonomous, and self-referential entity, but rather to surrender to the greater reality, relinquishing the illusion of separation and embracing the unitive consciousness that pervades all being. *The Art of Being and Becoming* by Khan (2005) is an example in the Sufi tradition that focuses more than usual on self-reflection to discover one’s true self, but still ultimately conceives of everyone’s true self in a somewhat prescribed spiritual way.

## Author’s note

Mohsen Joshanloo wrote the first draft. Mohsen Joshanloo and Dan Weijers finalized the manuscript. Dan Weijers led the revision of the draft in light of reviewers’ comments. Both authors read and approved the final manuscript.
